# Centralized daily drug management in clinical trial institutions and specific implementation plans

**DOI:** 10.3389/fdsfr.2025.1620314

**Published:** 2025-06-20

**Authors:** Yu Tang, Xiaolong Fang

**Affiliations:** ^1^ School of Medical, Jiangnan University, Wuxi, China; ^2^ Department of General Surgery, The Affiliated Wuxi No. 2 People’s Hospital of Jiangnan University, Wuxi, China; ^3^ Drug Clinical Trial Facility, Affiliated Hospital of Jiangnan University, WuXi, China

**Keywords:** clinical trials, drug management, implementation plan, standardization, centralized management mode

## Abstract

**Background:**

Experimental drug management is an important part of clinical trial management and directly relates to the reliability of clinical trial data and the safety of participants. This study explores the implementation of centralized pharmacy management in clinical drug trials, addressing the limitations of decentralized approaches.

**Methods:**

It involves the establishment of a centralized drug warehouse with unified processes and digital systems, primarily relying on the Cold Chain Cloud Platform, Service Cloud Platform, and Enterprise WeChat Platform, to enhance precision and efficiency.

**Results:**

It indicated a significant reduction in error rates and improved adherence to clinical trial protocols. The centralized model also minimized protocol deviations and strengthened participant safety.

**Conclusion:**

It highlights the benefits of centralized management in enhancing drug administration efficiency and maintaining high standards of trial conduct, suggesting future integration with Artificial Intelligence (AI) and Electronic Health Records (EHR) for more precise pharmaceutical services.

## 1 Introduction

In recent years, the decentralized approach to managing investigational drugs has demonstrated significant limitations (National Medical Products Administration, 2020a; National Medical Products Administration, 2020b), such as difficulties in coordination between departments, which reduces efficiency. In contrast, institutional centralized pharmacy management is characterized by its efficiency and standardization. Therefore, centralized pharmacy management is positioned as an emerging trend that is expected to enhance the safety of drug quality, storage, and utilization. Effective drug management throughout clinical trials is integral to the progression and precision of trial outcomes, influencing both data accuracy and reliability.

This significance is reflected in standards such as the current *Good Practice for the Quality Management of Pharmaceutical Clinical Trials in China (No. 57 of 2020)* and the *ASHP Guidelines for the Management of Investigational Drug Products* (Feng et al., 2020; Guo et al., 2019). As regulatory frameworks evolve with increasingly comprehensive drug management criteria, drug administrators must navigate these requirements to ensure accurate, complete, and practical application in daily operations while minimizing error risks.

Centralized drug warehouse management, employing unified processes, standardized operations, and digital information systems, enhances precision and efficiency across experimental drug storage, distribution, and retrieval, thereby raising overall management quality and productivity in drug logistics (Mohammad et al., 2021; Dagenais et al., 2022; Brodie et al., 2019). This centralized framework further minimizes error rates, safeguards participant welfare, and strengthens adherence to clinical trial protocols (Jones et al., 2020).

Effective management of investigational drugs is integral to ensuring the scientific integrity, accuracy, and impartiality of clinical trial outcomes (Sessler and Myles, 2020). With regulatory management on clinical trial drugs becoming increasingly rigorous and standardized, monitoring drug management practices has emerged as a core aspect of the on-site evaluation of trial institutions and project verifications (Spira et al., 2021; Thall et al., 2023). Currently, however, the regulatory framework lacks specific guidelines on investigational drug management, allowing institutions to establish tailored protocols aligned with hospital-specific needs (Magnuson et al., 2021). Irrespective of the chosen approach, adherence to Good Clinical Practice (GCP) standards remains paramount to affirming the scientific validity, authenticity, and reliability of clinical trial data.

Drug clinical trial institutions in China primarily implement three management models: centralized management, decentralized management, and a collaborative approach between institutions and specialized teams (hybrid management) (Gulden et al., 2019; Calabrese et al., 2023). There is a scarcity of international research specifically exploring the centralized management of drugs in clinical projects. Currently, centralized drug management is advantageous for the summarization of drug resources and the enhancement of management efficiency. In contrast, decentralized drug management can refine functions, including managing specific drugs at the department level, ensuring timely delivery to participants, and reducing data deviations in clinical projects caused by the drug transportation process (Thomas et al., 2020). This model may have advantages in flexibility and response speed, but it could face challenges in standardization and coordination. The hybrid model combines the strengths of both centralized and decentralized approaches, but it may increase management complexity.

At present, some institutions are plagued by chaotic personnel management, slow project progress, and inadequate drug monitoring (Fan et al., 2022). In contrast to other institutions, our hospital undertakes a larger number of projects and involves a broader range of departments. By leveraging a centralized pharmacy management platform to integrate project management, execution, and supervision, we have achieved smoother project progress and more standardized drug management. The application of this management platform effectively simplifies actual processes and maximizes the utilization of resources.

The centralized pharmacy of our hospital relies on a variety of digital platforms (Table 1) and adopts a modular design. Each module can be flexibly adjusted and expanded according to specific tasks. This design not only enhances the adaptability of the system, but also facilitates subsequent optimization and upgrades. In contrast, some existing models often have a fixed architecture, which makes it difficult to make effective adjustments for different tasks.

**TABLE 1:** Digital Tools and Platforms Summary.

Digital tool or platform name	Function description	Version data	Update frequency	Access permission	Supplier information
Cold Chain Cloud Platform	Mainly records the real-time temperature during the transportation and storage process of pharmaceuticals	V3.0	Updated every hour	Limited to authorized users in transportation and central pharmacy	Hangzhou Zeda Instrument Co., Ltd.
Service Cloud Platform	Mainly introduces the number of equipment in the current laboratory, online quantity, offline quantity, abnormal alarm quantity, and the location of the laboratory	V3.0	Updated daily	Limited to authorized users in the central pharmacy	Hangzhou Zeda Instrument Co., Ltd.
Enterprise WeChat Platform	Mainly includes communication and writing between hospitals, institutions, and enterprises; management and statistics of file, equipment, and temperature recording information	4.1.36.6011	Real-time update	Internal employees of the enterprise	Shenzhen Tengxun Computer System Co., Ltd.

This table outlines three digital platforms used in pharmaceutical management. The Cold Chain Cloud Platform (V3.0) tracks real-time temperatures during transportation, updated hourly, and is accessible to authorized users from Hangzhou Zeda Instrument Co., Ltd. The Service Cloud Platform (V3.0) monitors equipment and inventory, updated daily, with access limited to the central pharmacy’s authorized users. The Enterprise WeChat Platform (4.1.36.6011) facilitates communication and management within the company, with updates in real-time, accessible to all internal employees, provided by Shenzhen Tengxun Computer System Co., Ltd.

In November 2020, Our hospital utilizes a centralized management framework, establishing an independent GCP central pharmacy staffed with dedicated pharmacists to manage clinical trial drugs. This model satisfies specific regulatory demands for inventory, secured storage, controlled access, registration, and prescription management. Additionally, a dedicated dispensing window for clinical trial drugs is established. This minimizes issues such as substandard storage, irregular distribution and usage, and incomplete documentation (Liu et al., 2020; Lagreula et al., 2021; Cao et al., 2023). This study presents critical aspects of daily central warehouse management alongside a targeted implementation strategy.

## 2 Regulatory context

In China, the regulatory standards, including the *Drug Clinical Trial Quality Management Standards* and the General Rules, impose strict requirements on the management of investigational drugs and clinical trial data (Administration, 2004; Omidian and Omidi, 2022). They mandate detailed protocols for handling drugs and emphasize meticulous documentation practices. These regulations also outline essential qualifications for trial personnel and standards for pharmacy management and infrastructure (Cao et al., 2022; Lin et al., 2020; Tang et al., 2021). Furthermore, the *Requirements for Drug Records and Data Management* and *the Key Points and Judgment Principles of Drug Registration Verification* reinforce the need for stringent management practices and controlled documentation procedures (Wei et al., 2021; Zhou et al., 2022).

China’s GCP regulations are developed based on the Food and Drug Administration (FDA) and European Medicines Agency (EMA) regulations with localization modifications. In terms of the preparation of investigational medicinal products (IMPs), all three sets of regulations require that the preparation of IMPs should comply with the relevant requirements of Good Manufacturing Practice (GMP) for clinical trial medicinal products. The packaging and labeling should indicate that the products are for clinical trial use only and provide information on the clinical trial and the IMPs (Boesen et al., 2021).

Regarding the management of IMPs, the approval authorities differ among the three (Chiodin et al., 2019). China’s regulations require the sponsor to be responsible for providing IMPs to investigators and clinical trial institutions. IMPs shall not be provided before obtaining approval or filing from the ethics committee and the drug regulatory authority. The FDA requires the sponsor to provide IMPs and prohibits the provision of IMPs before obtaining approval from the IRB and the FDA. The EMA requires the sponsor to provide IMPs and prohibits the provision of IMPs before obtaining approval from the ethics committee and the EMA.

## 3 Drug management measures for clinical trials

**FIGURE 1:**
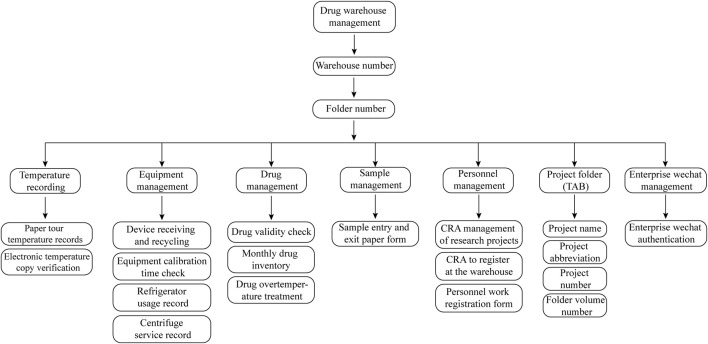
Classification and Coding Guidelines for Drug Folders. The drug warehouse document management flowchart is comprehensively structured, detailing six core areas: temperature monitoring, equipment management, drug inventory, sample tracking, personnel records, and project documentation. Temperature monitoring includes both paper-based and digital records. Equipment management encompasses documentation related to equipment acquisition, recycling, and utilization. Drug inventory management involves tracking stock levels and expiration dates. Sample tracking records each sample’s entry and exit from the warehouse. Personnel records document CRA and CRC registrations, while project documentation maintains an organized record of project names and identification numbers.

Centralized clinical drug management mainly consists of the following parts: personnel management, file management, appropriate drug storage conditions, complete temperature warning mechanism, systematic drug double-check system and sharing of medication files (Figure 1) (Table 2).

**TABLE 2:** Pharmacy Centralization Management Regulations and the Corresponding Laws.

Centralized pharmacy management regulations	Relevant regulatory provisions	Main content
Personnel Management - Drug Managers	“*Guide to the Management of Drug Clinical Trial Institutions*” P53	1. The clinical trial pharmacy should be staffed with 2–3 professionals with a pharmacist or higher qualification as drug managers 2. All personnel responsible for the management of clinical trial drugs must undergo GCP training, pass the assessment to obtain a certificate, and be authorized by the principal investigator
Personnel Management - CRC Qualifications	*Drug Clinical Trial CRC Management - Guangdong Consensus* (2024 Edition)	1. Clinical trial institutions should verify the qualifications of each project’s CRC. This verification includes checking whether the CRC’s affiliated SMO has signed a tripartite agreement with this site, and whether there is a letter of appointment from the SMO company specifically for this trial project
Personnel Management - CRA	*GCP* Article 49, Section 50	1. Investigators appointed by sponsors should receive appropriate training, possess the necessary knowledge of medical and pharmaceutical clinical trial supervision, and be able to effectively perform supervisory duties
Document Management - Document Records	*GCP General Principle* Article 7	1. All paper or electronic documents of clinical trials should be properly recorded, processed, and stored to accurately explain and confirm. The privacy of participants and related information should be protected
Document Management	GCP Chapter 8, Article 78, Section 79	1. Essential documents are those that sponsors, regulatory authorities inspect clinical trials, and confirm the authenticity and completeness of clinical trial implementation and data collection 2. Sponsors, investigators, and clinical trial institutions should confirm that there are places and conditions for storing essential documents of clinical trials. The conditions for storing documents should prevent direct exposure to light, moisture, fire, etc., which are conducive to the long-term preservation of documents
Suitable Drug Storage Environment	*Drug Clinical Trial CRC Management - Guangdong Consensus* (2024 Edition)	1. Institutions should establish an independent clinical trial pharmacy with a reasonable layout and sufficient space to meet the storage requirements of trial drugs. The area should be clearly divided by function, including receiving area, dispensing area, storage area, and return area
Standard Drug Storage Equipment	*GCP* Article 5	1. Trial drugs must be stored independently in a dedicated pharmacy, equipped with required devices (such as refrigerators, constant temperature cabinets)

This table maps personnel management, document management, and drug storage management in centralized pharmacies to their corresponding regulations, focusing on detailed provisions within specific laws.

### 3.1 Personnel management

To comply with legal and regulatory standards, the central pharmacy requires the appointment of at least two drug administrators who are qualified in GCP. These administrators must have expertise in the clinical trial drug management system and be proficient in Standard Operation Procedures (SOPs) to ensure effective drug management (Zhang et al., 2020). Alternatively, these administrators must undergo annual GCP training and pass an accredited state-recognized assessment to maintain valid GCP certification (Miyauchi et al., 2022).

The study systematically examines the qualifications of clinical research coordinators (CRCs) across programs, focusing on whether affiliated Site Management Organization (SMO) companies have formalized tripartite agreements with sites and provided delegation letters specific to test projects. The audit further verifies the validity of CRCs’ IDs, the presence of updated GCP training certifications, institutional work numbers, and Principal Investigator (PI) authorization. Only CRCs meeting these approval standards may engage in authorized drug management activities, including drug verification, transport, temperature monitoring, and end-of-month inventory management (National Medical Products Administration, 2020b).

The study further assesses the credentials of clinical research associates (CRA), examining whether each CRA is affiliated with the sponsor or the appointed Contract Research Organization (CRO), holds an officially endorsed letter of appointment, and has been formally registered with the organization, including the acquisition of a CRA job number. Access to the drug supervision center’s storage facilities, as well as drug receipt and recycling areas, is restricted to CRAs meeting all qualification requirements.

### 3.2 File management

#### 3.2.1 Controlled version and number of files

The Center establishes SOPs (JG-SOP-V2.0) governing the control of drug-related documents, designating the drug administrator as the sole recipient for these materials. Each clinical project’s CRA customizes the drug documents per the hospital’s template, followed by review and approval from the project team, PI, and Quality Assurance. After verification, the documents are printed, handed over to the drug administrator, and then uniformly filed into the project folder upon document retrieval and project completion. During trials, when updates to controlled documents are required or existing copies are exhausted, additional documents are issued. The CRA submits an application via the platform, secures approval for the updated or new documents, and delivers them to the drug administrator, completing the official record.

#### 3.2.2 Record of files

Documentation must remain accurate, prompt, and comprehensive. Forms maintained by the center encompass drug inventory logs, participant drug issuance and retrieval records, transportation temperature logs, participant drug receipt forms, and storage temperature logs. Any irregularities during storage, such as temperature deviations, drug loss, or delivery discrepancies, must be promptly documented, with a monthly inventory check to confirm drug stocks. Additionally, GCP centralized pharmacy management requires three primary types of documentation: electronically derived temperature records indicating storage conditions (and their copies); logs for receiving, calibration, recovery, and maintenance of refrigerators, incubators, thermometers, and similar equipment; and registration records of CRA monitoring and site visits.

#### 3.2.3 Folder management

Establishing standardized coding rules for folders optimizes file placement and retrieval processes. Folder storage must be systematically organized within designated cabinets, with storage spaces engineered for fire, theft, insect, and light protection (Myles, 2022). Additionally, folder locations should be dynamically realigned according to project progress. Implementing a rigorous approval process for folder access, borrowing, and return is essential to safeguard file security and confidentiality, restricting access solely to authorized personnel. Together, these measures promote uniform folder storage, expedite file retrieval, and ensure the authenticity and completeness of records, preserving both the integrity of documentation and the confidentiality of participants’ drug use data.

### 3.3 Appropriate drug storage conditions

#### 3.3.1 Appropriate drug storage partitions

At the drug warehouse, areas for qualified, unqualified, and holding drugs are marked with colored bands. Expired and empty packages are stored in the nonconforming area, while overtemperature drugs are held pending review. Regular checks of expiration dates and temperatures are crucial for proper storage. Sections for expired drugs and packaging, along with isolation fridges and recycling bins, ensure timely disposal of unusable drugs.

#### 3.3.2 Standard drug storage equipment

Effective GCP management mandates air conditioning, dehumidifiers, and humidifiers within drug storage areas to maintain requisite environmental conditions, with all equipment verified for proper function and supported by valid calibration certificates. A dynamic refrigerator information log should comprehensively record equipment details—serial numbers, storage durations, suppliers, calibration expiration dates, and sponsor retrieval dates. Regular monitoring of calibration status is essential, with timely notifications to ensure prompt professional recalibration. Annual calibration also necessitates temperature-monitoring tools, such as thermometers and temperature cards, accompanied by an electronic calibration certificate. Additionally, a dynamic log for temperature-monitoring equipment captures critical information, including serial numbers, storage timelines, providers, certificate validity, and sponsor retrieval schedules. Thermometers approaching calibration expiration must be promptly replaced, ensuring accuracy of exported temperature records.

### 3.4 Complete temperature warning mechanism

**FIGURE 2:**
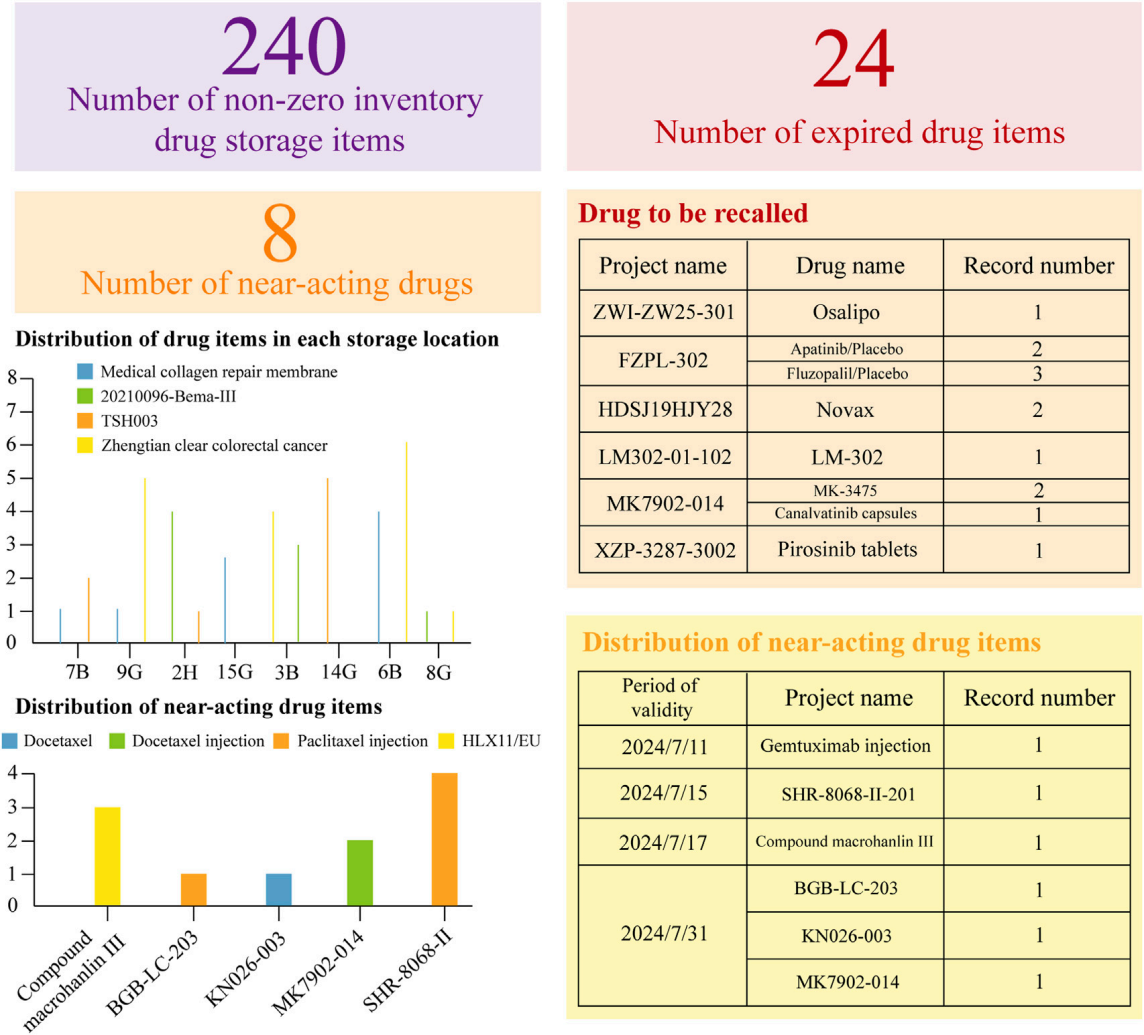
Dynamic Inventory Management Table for Clinical Trial Drugs. This statistical table is based on the WeChat Work platform. It has organized the current expiration dates of the drugs, providing a clear overview of their validity. Additionally, it has meticulously listed all the relevant drugs, ensuring a comprehensive inventory. Moreover, the table has accurately specified the storage locations for each drug, facilitating efficient management and easy access.

Initially, a dynamic drug inventory table is created, followed by the development of a dial based on this table to provide a clear visual representation of current drug inventory levels, as illustrated in Figure 2. This figure details the quantities of drugs nearing expiration or already expired, alongside their respective project names and precise expiration dates. By selecting a specific number within the item column, users can access a detailed breakdown of each drug in stock.

**FIGURE 3:**
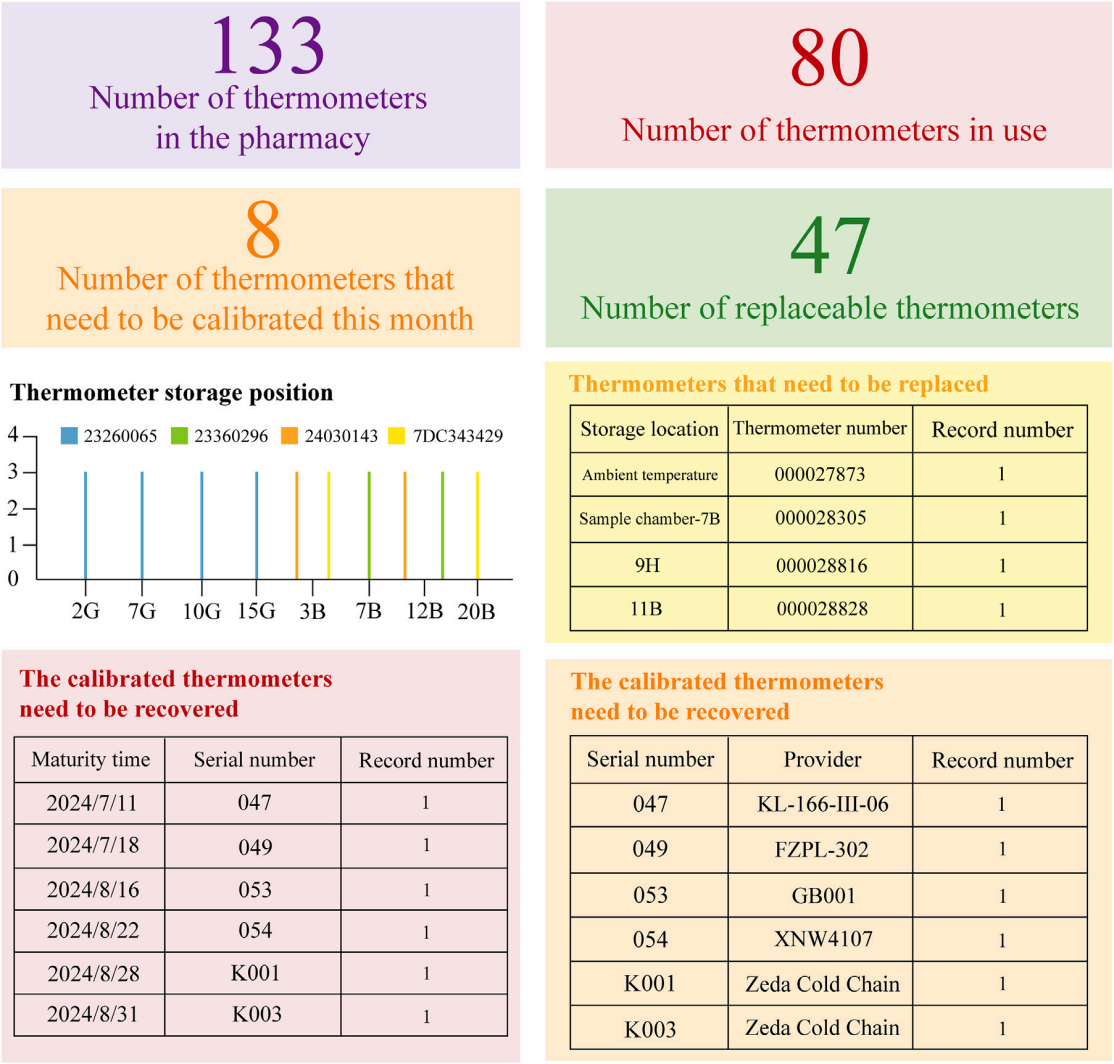
Dynamic Inventory Management Table for Temperature Recording Equipment. Based on the WeChat Work platform and Cold Chain Cloud Platform (www.zjueecloud.com), this dynamic table compiles extensive statistical data on the inventory of pharmacy thermometers. It includes information on their validity period, designated uses, and suppliers for each unit. Additionally, it provides a statistical analysis of the number and distribution of thermometers that are nearing their expiration dates.

In the second place, the instrument panel incorporates dynamic thermometer tables and temperature cards to precisely represent the status of temperature monitoring equipment within the pharmacy. As illustrated in Figure 3, to assess the need for advance procurement or replacement with a newly calibrated thermometer supplied by the project team, the panel tracks metrics including the total thermometer inventory, active thermometers, replacement units, and thermometers due for calibration each month. Additionally, the dynamic table records both the quantity and provider of each thermometer, enhancing traceability and enabling prompt recovery and replacement in case of malfunction. Selecting specific color-coded indicators (e.g., “35 thermometers due for calibration this month”) reveals detailed equipment information and current status.

**FIGURE 4:**
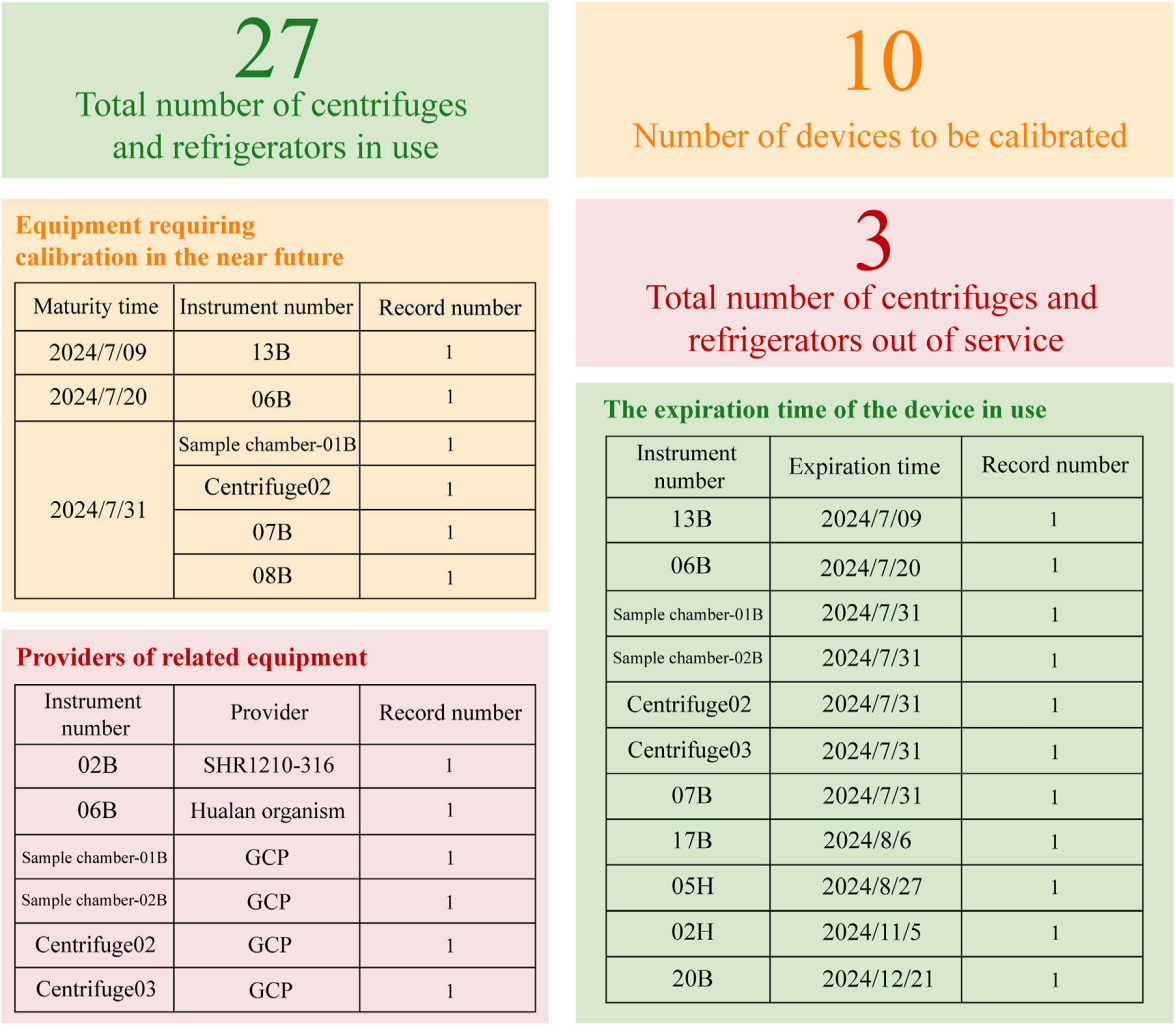
Dynamic Inventory Table for Refrigerators and Centrifuges. Based on the WeChat Work platform, this dynamic inventory table provides a clear overview of the equipment status, categorizing items into active, inactive, or those requiring calibration. It also details the calibration due dates for each item and includes supplier information related to specific equipment.

Finally, the institution’s centralized sample processing method mandates unified management of equipment, including centrifuges and ultra-low temperature refrigerators. The dashboard, illustrated in Figure 4, is structured according to real-time equipment monitoring tables, detailing the total number of active centrifuges and refrigerators, units requiring calibration, and those marked as out of service. Additionally, the dynamic table delineates storage locations, assigned units, and precise calibration expiration dates. This configuration enables drug administrators to readily monitor equipment in the center’s drug warehouse, providing immediate access to usage, maintenance, and calibration details, thereby enhancing the robustness of the drug management system.

By developing and refining diverse dynamic charts and dashboards, enhanced management of drug and thermometer expiration dates, as well as calibration validity for related equipment, can be achieved. This approach ensures that clinical trial drugs are consistently maintained within appropriate storage conditions, thereby supporting rigorous drug management standards. These measures are designed to prevent the distribution of expired medications to participants, safeguarding their safety and wellbeing.

### 3.5 Systematic drug double-check system

The pharmacy at the Center has adopted a dual verification system for a closed-loop management process covering drug reception, storage, distribution, return, and recovery. Two administrators and a CRC or CRA verify all incoming drugs’ documentation, temperatures, calibration certificates, and quality reports, ensuring they match delivery and application data. Daily temperature checks and monthly inventory reconciliations are conducted to confirm inventory accuracy against both paper and digital records.

For drug distribution, two administrators verify prescriptions and, if necessary, randomization orders before dispensing medication, both signing to minimize errors. Some procedures require photographic evidence for comprehensive project team review. Unused drugs and packaging are returned by CRCs to administrators for inventory checks and recorded details (Nicholson, 2023).

Expired drugs, returned unused drugs, and used packaging are systematically recorded and verified by CRAs and administrators. The CRA also files a recovery application, uploading electronic documents and archiving originals securely. This dual-core system ensures a fully compliant, closed-loop process, minimizing error risks.

### 3.6 Sharing of medication files

The Center Pharmacy’s network platform facilitates secure, encrypted file exchanges with CRCs and CRAs, enhancing efficiency. It shares calibration certificates and monthly temperature records for warehouse equipment, alerts CRAs to expiring drugs via a dynamic table, and provides device-specific information for calibration and recycling. Electronic inventory ledgers grant CRCs access to monitor drug levels and flag expiring items. A mind map of the warehouse process is shared to ensure clear protocol understanding, reduce miscommunication, and boost workflow efficiency.

## 4 Outcomes

Centralized drug management has reduced the error rate in management. In the baseline phase, the most common type of error was incorrect dosing time, with an error rate of 17.6 per 100 doses. After the intervention, the error rate for incorrect dosing time significantly decreased from 17.6 to 9.8 (Table 3). Protocol deviations are mainly classified into five levels: Level 1 has no impact on data quality or subject safety; Level 2 has a minor impact on data quality; Level 3 has a minor impact on subject safety; Level 4 has a significant impact on data quality or subject safety; Level 5 results in subject death. Protocol deviations are common in clinical trials and need to be reduced through strict drug management and monitoring. The classification and handling measures of deviations are crucial for ensuring the scientific nature of the trial and the safety of subjects. According to the research, the severity of deviations is significant for guiding the handling and prevention of deviations (Table 4).

**TABLE 3:** Comparison of Error Rates Before and After the Implementation of Centralized Clinical Drug Management.

Error type	Baseline (pre-centralization) error rate (%)	Centralized error rate (%)	Change (post-intervention) (%)
Incorrect Adminstration Time	17.6	9.8	−7.8
Incorrect Solvent/Dilution Volume (Injection)	11.0	2.7	−8.3
Incorrect Dosage	3.0	1.9	−1.1
Incorrect Drug	1.0	0.7	−0.3
Incorrect Administration Route	0.8	0.2	−0.6

This table illustrates the reduction in medication administration error rates following the implementation of a centralized system. The centralized error rates are significantly lower than the baseline rates for all types of errors.

**TABLE 4:** Comparison of Protocol Deviations Before and After the Implementation of Centralized Clinical Drug Management.

Deviation level	Baseline (pre-centralization) deviation (%)	Post-centralization deviation (%)	Change (post-intervention) (%)
Level 1	65.3	81.6	16.3
Level 2	20.5	15.3	−5.2
Level 3	12.5	3.1	−9.4
Level 4	1.7	0.0	−1.7
Level 5	0.0	0.0	0.0

This table shows the impact of centralization on deviation levels in a process. Level 1 deviations increased by 16.3% post-centralization, while Level 2, 3, and 4 decreased by 5.2%, 9.4%, and 1.7% respectively. Level 5 remained unchanged at 0%.

## 5 Limitations

The centralized drug management system exhibits certain limitations, given the late establishment of the dedicated team and ongoing adaptations to the standards set forth by the National Bureau of Drug Clinical Trial Institutions Supervision and Inspection Points and Judgment Principles (Trial). Firstly, some members may lack sufficient experience in clinical trials. This could lead to errors in trial design, execution, and management. To mitigate this potential risk, our institution regularly organizes standardized clinical trial training sessions. After the training, participants are assessed, and we also arrange for team members to visit and exchange experiences with more established teams.

Secondly, with limited clinical project resources, our newly established team may face constraints in terms of funding, equipment, and human resources, which could affect the quality and speed of trials. In this regard, our hospital actively recruits advanced medical teams from home and abroad to provide technical support for clinical project recruitment and implementation. In terms of publicity, we collaborate with higher-level management departments and lower-level community hospitals to popularize clinical trial projects among the public. This helps them gain a basic understanding of these projects and provides a pool of potential participants for future clinical trials.

Thirdly, in terms of regulatory compliance, new teams may face the challenge of quickly adapting to and complying with the constantly changing clinical trial regulations and guidelines. This requires team members to keep up with the times, continuously track the latest international information on clinical trial projects, and integrate it with domestic regulations and guidelines to form a theoretical system suitable for our hospital’s institution.

Lastly, regarding technological adaptability, new institutions may need time to adapt to and adopt the latest clinical trial technologies and methods. This demands that we stay at the forefront of international technology. For example, by employing artificial intelligence and machine learning, AI technology can be utilized in clinical trial data management for data collection and integration, data cleaning and standardization, data monitoring and quality control, as well as data analysis and prediction. I believe that these limitations and the lessons learned from them can make our drug clinical projects more standardized and efficient.

## 6 Discussion and conclusion

**FIGURE 5:**
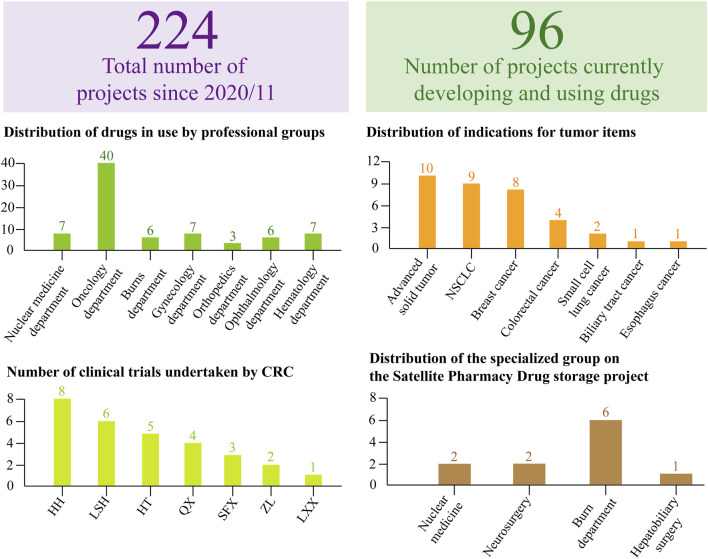
Dynamic Inventory Table for Clinical Trial Projects. Based on the WeChat Work platform and Service Cloud Platform, the dynamic project table provides a structured overview of total projects and those actively in development. For projects currently under investigation, statistical analysis includes the distribution across hospital professional groups, specific indications per project, and assigned personnel managing both the projects and corresponding sample processing. This streamlined approach to data and process management supports the efficient completion of tasks, ensuring both quality and quantity.

Since November 2020, the center has managed 224 clinical trial projects (Figure 5), with 96 currently in active research stages requiring ongoing liaison with drug regulatory authorities. Among these, 49 projects constitute the primary workload. Specialized trials, including oncology and pediatric endocrinology, are characterized by extended timelines, complex treatment protocols, and elevated incidences of adverse and serious adverse events. The center’s drug storage facility maintains a substantial inventory, necessitating regular calibration and upkeep of numerous devices, alongside extensive management of both handwritten and electronic records. Beyond documentation duties, the drug administrator oversees meticulous logging and maintenance of equipment—such as thermometers and refrigerators—including details on acquisition, servicing, calibration, and eventual decommissioning, while coordinating with professionals for on-site calibration as needed. Daily temperature monitoring is recorded on controlled temperature sheets, with all data exported, securely encrypted, and uploaded to the platform monthly for secure sharing. The two drug administrators further manage daily operations encompassing drug receipt, maintenance, inventory control, distribution, and recycling.

In terms of the costs associated with the construction of a centralized pharmacy, initially, new employees require training on standard operating procedures and other protocols. This involves expenses for training materials, venue rentals, and instructor fees. To attract and retain high-quality talent, a competitive compensation system must be in place to ensure team stability and professionalism. Additionally, there are costs for purchasing hardware such as servers, network equipment, and security devices. Factors such as brand, performance, and capacity of the equipment need to be considered. During the use of these devices, regular maintenance and potential replacement due to technological advancements are necessary, incurring additional costs. Furthermore, the purchase of commercial software, including operating systems and database software, entails software licensing fees and customization costs.

Regarding the benefits of a centralized pharmacy, professional operation and maintenance by personnel, along with robust equipment protection, help prevent security incidents such as data breaches, tampering, and loss. This reduces the risk of system crashes due to vulnerabilities or equipment malfunctions, thereby maintaining the normal operational order of the pharmacy. Workflow automation through software tools enhances work efficiency. Moreover, coordinated management of personnel, equipment, and software leads to efficient resource utilization (Table 5).

**TABLE 5:** Evaluate the strengths and weaknesses of each management component using a SWOT analysis.

Management component	Strengths	Weaknesses
Personnel Management	Standardized processes for personnel entry and exit ensure project responsibilities are assigned to each person	Frequent personnel changes may lead to insufficient work handovers and content confusion
Document Management	Systematic clinical trial document recording and storage comply with GCP regulations for protecting original drug data	If the document management system is not updated in time, there may be risks of data exposure
Drug Management	Use of advanced drug storage technology and standardized alarm mechanisms comply with GCP storage conditions for drugs	Drug storage facilities require high costs, and system failures may lead to temperature fluctuations in drug storage
Drug Double-check System	Ensures the accuracy and traceability of drug management to prevent errors and omissions, thereby ensuring the quality and safety of clinical trials	There is still a possibility of errors occurring
Document Sharing	Improves work efficiency, enhances collaboration between enterprises, hospitals, and centralized pharmacies, and increases transparency of work processes	May increase the risk of data exposure and loss

Explore the advantages and disadvantages of various management modules, focusing specifically on personnel management, document management, drug management, the drug double-check system, and document sharing. Each aspect will be analyzed to understand its specific strengths and weaknesses, providing a comprehensive evaluation of these management components.

Since November 2020, the clinical trial center has been subjected to five verifications by the National Bureau, with no corrective actions reported for drug or sample management. This outcome directly reflects the National Bureau’s acknowledgment of the Center’s standards in these areas. In practice, the centralized management model has significantly improved drug administration efficiency, minimized protocol deviations, and strengthened participant drug safety.

While centralized drug management provides considerable benefits, satellite pharmacies remain integral within the four specialized groups due to unique protocol demands, including requirements for intraoperative or emergency drug administration for specific projects (Song and Kim, 2020). However, management of thermometers, refrigerators, drug expiration management, as well as drug receipt and retrieval processes in these satellite pharmacies, is collaboratively maintained by the Professional Group and the central pharmacy’s drug manager.

Looking to the future, the development direction of clinical centralized pharmacies will shift towards the integration of AI and EHR (Kabella et al., 2025; Shermock et al., 2023). By integrating the centralized pharmacy system with EHR, comprehensive sharing and integration of patient medical information can be achieved. This allows for a more thorough understanding of patients’ conditions and medication use, enabling the provision of more precise pharmaceutical services. Based on the integrated EHR data, AI can offer pharmacists more accurate decision support for medication use. For instance, by analyzing patients’ medical history, genetic information, and current conditions, AI can predict their reactions to certain medications, assisting pharmacists in selecting the most appropriate drugs and dosages to improve treatment outcomes.
